# A Chemosensory GPCR as a Potential Target to Control the Root-Knot Nematode *Meloidogyne incognita* Parasitism in Plants

**DOI:** 10.3390/molecules24203798

**Published:** 2019-10-22

**Authors:** Emmanuel Bresso, Diana Fernandez, Deisy X. Amora, Philippe Noel, Anne-Sophie Petitot, Maria-Eugênia Lisei de Sa, Erika V. S. Albuquerque, Etienne G. J. Danchin, Bernard Maigret, Natália F. Martins

**Affiliations:** 1 Université de Lorraine, CNRS, Inria, LORIA, F-54000 Nancy, France; emmanuel.bresso@loria.fr (E.B.); philippe.noel@inria.fr (P.N.); 2 EMBRAPA Genetic Resources and Biotechnology, Brasilia 70770-917, DF, Brazil; diana.fernandez@ird.fr (D.F.); deisyamora@yahoo.com.br (D.X.A.); eugenialisei@gmail.com (M.-E.L.d.S.); erika.albuquerque@embrapa.br (E.V.S.A.); 3 IRD, CIRAD, Université de Montpellier, IPME, F-34398 Montpellier, France; anne-sophie.petitot@ird.fr; 4 INRA, Université Côte d’Azur, CNRS, Institut Sophia Agrobiotech, F-06903 Sophia-Antipolis, France; etienne.danchin@inra.fr

**Keywords:** *Meloidogyne incognita*, homology modelling, molecular dynamics, virtual screening

## Abstract

Root-knot nematodes (RKN), from the *Meloidogyne* genus, have a worldwide distribution and cause severe economic damage to many life-sustaining crops. Because of their lack of specificity and danger to the environment, most chemical nematicides have been banned from use. Thus, there is a great need for new and safe compounds to control RKN. Such research involves identifying beforehand the nematode proteins essential to the invasion. Since G protein-coupled receptors GPCRs are the target of a large number of drugs, we have focused our research on the identification of putative nematode GPCRs such as those capable of controlling the movement of the parasite towards (or within) its host. A datamining procedure applied to the genome of *Meloidogyne incognita* allowed us to identify a GPCR, belonging to the neuropeptide GPCR family that can serve as a target to carry out a virtual screening campaign. We reconstructed a 3D model of this receptor by homology modeling and validated it through extensive molecular dynamics simulations. This model was used for large scale molecular dockings which produced a filtered limited set of putative antagonists for this GPCR. Preliminary experiments using these selected molecules allowed the identification of an active compound, namely C260-2124, from the ChemDiv provider, which can serve as a starting point for further investigations.

## 1. Introduction

Root-knot nematodes (RKNs) seriously threaten global food production: these microscopic, soil-dwelling worms are highly destructive and cause up to 100% yield loss in important life-sustaining crops like soybean, rice, cotton, tomato, etc. [1,2,3]. Their destructive capacities are due to their ability to infect roots and cause galls (swellings or knots) in their host plants. The nematodes feed and develop in the galls, in the process impeding nutrient and water uptake by the host plant resulting in poor growth and crop yield. Additionally, such damages increase the severity of opportunistic infections by other soil pathogens [4].

Conventional control methods, including crop rotation, use of resistant cultivars and biological control have limited efficacy or are not available against RKNs that have a wide host range [5]. Traditionally, RKN management relied heavily on carbamates, organophosphates and fumigant nematicides. Due to environmental, health and safety concerns, the most efficient chemicals to control nematodes are no longer used and are now being withdrawn [6]. As such, there is an urgent need for safer and sustainable solutions to control RKNs. Currently, several development trials are being undertaken around the world to find and develop eco-friendly nematicides [7,8]. On this line of research, several new compounds are proposed but their environmental safety remains to be validated [9,10].

In recent years, and since the availability of several nematodes genomes [11], bioinformatics analyses have provided the basis to explore specific ways to control RKNs invasion by targeting specific genes implied in plant parasitism [12]. A promising approach would consist in interfering specifically with the olfactory perception of plant chemicals that attract nematodes, or with their developmental cycle into the host plant [13,14,15]. These strategies would enable protection of the plants against infection by root-knot nematodes without altering other environmental characteristics. From a scientific point of view, such researches provides new insights towards linking molecular methods with biochemical processes for plant protection against RKNs.

In the rice RKN (*Meloidogyne graminicola*), the disruption of neuropeptides involving G protein-coupled receptors (GPCRs) signalling disturbs both behavior and migration abilities infection [16]. This recent discovery offers a promising possibility to control the behavior of this parasite as well as other RKN such as *Meloidogyne incognita* which is amongst the most devastating plant parasite worldwide [17]. As GPCRs are recognized as an important target family for many pharmaceutical developments [18], we prioritized the identification and classification of GPCRs from *M. incognita* in order to help control this agricultural pest. GPCRs constitute a superfamily of transmembrane proteins acting as receptors that sense molecules and trigger transduction pathways in cells. Their 3D structure is highly conserved in opposition to their amino acid sequences that are poorly conserved. Most GPCRs have are 200–1000 amino acids long, show a 7 transmembrane helices structure and several additional domains related to their functional specificity and useful for their classification.

In this paper, we mined the ensemble of predicted proteins of the RKN *M. incognita* to identify putative GPCRs and select the most likely to interfere with the parasite life cycle. We then built a 3D model of this receptor to perform a receptor-based in silico virtual screening for identification of chemical compounds able to interfere specifically with this GPCR. Finally, we validated the activity of the compounds through preliminary tests in vivo in order to establish the proof-of-concept to our strategy.

## 2. Results

### 2.1. GPCR Identification and Selection

**Figure 1:**
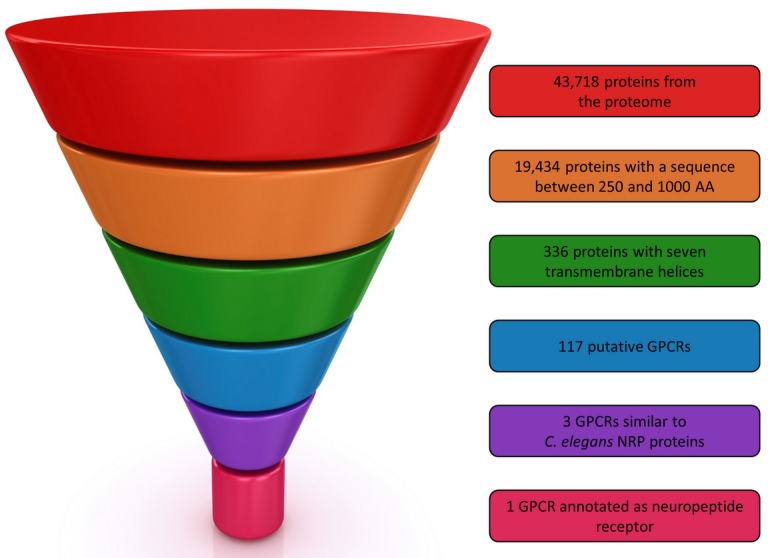
G protein-coupled receptors (GPCRs) selection procedure.

From the 43,718 predicted proteins of *M. incognita* 19,434 had a length between 250 and 1000 residues (Figure 1). Next, we retained only those with seven predicted transmembrane helices. The 336 selected proteins were finally submitted to GPCRpipe in order to predict putative GPCRs. As a result, 117 proteins can be considered as putative GPCRs in *M. incognita*. In order to validate our procedure, we checked that the only protein found in UniProt described as a “GPCR” in *M. incognita*, namely Q2TGX5, was in the list of the 117 proposed GPCRs.

To select among these 117 putative GPCR, the most suitable ones for our drug design campaign, we mined the literature and all protein sequence databases to obtain additional information about putative nematode GPCRs. From this search, some important information was collected, especially concerning nematode chemosensory system [19] and neuropeptides GPCRs [20]. For instance, the mechanosensory role described in FMRFamide-like peptides family in several nematodes were particularly interesting [16,21,22,23,24,25,26] and this peptide family is also present in *M. incognita* [27]. The GPCRs associated to these peptides, such as the *C. elegans* flp-32 receptor [28] (uniprot G5EEB1) and FRPR-4 receptor [29] (uniprot A0A131MCZ4), have been shown to be involved in the regulation of sexual and locomotion behaviour of nematodes and are therefore interesting targets for inhibiting nematodes parasitism on plants. In this respect, NPR-1 receptor from *C. elegans* [30,31] (UniProt Q18534) and NPR-4 receptor from *C. elegans* and *Brugia malayi* [32] (Uniprot A0A078BS36 and A0A0J9XSQ0 respectively) were interesting targets for our purpose because the proteins were annotated as neuropeptide receptors. Consequently, we looked for their homologs among the 117 *M. incognita* putative GPCRs. Three proteins, Minc3s00126g05377, Minc3s01812g26474 and Minc3s00007g00462 respectively were found as NRP homologs from sequence analysis, but only the two proteins, Minc3s01812g26474 and Minc3s00007g00462 were annotated as “neuropeptide Y like GPCR” by InterProScan. The whole selection process is summarized in Figure 1.

### 2.2. Homology Modelling

**Figure 2:**
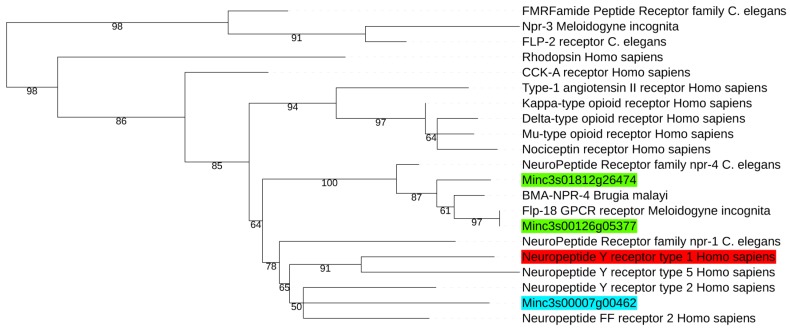
Phylogenetic tree obtained showing the proximity of Minc3s00126g05377 (green rectangle), Minc3s01812g26474 (green) and Minc3s00007g00462 (blue) with several GPCRs and especially with the neuropeptide Y Y1 receptor (red) for which a 3D structure was recently available in the PDB database. Branches with support value lower than 50 were colapsed.

The first step in homology modelling is to identify the most suitable templates to use to build the query 3D model. In our case, the best template should be a GPCR structure having sequence similarities with our query and belonging to the same GPCR category. In order to decide which one of the three possible GPCRs found above would be retained for performing the homology modelling step, we performed a phylogenetic analysis with several GPRCs with available 3D structures and others belonging to the neuropeptide receptors family. According to the phylogenetic tree obtained (Figure 2) it appeared that the protein Minc3s00007g00462 was the closest one to the *C. elegans* NPR-1 neuropeptide receptor (UniProt ID Q18534) and to the human neuropeptide Y Y1 receptor (UniProt ID P25929) corresponding to the PDB structure 5ZBH [33], while Minc3s00126g05377 and Minc3s01812g26474 were more related to the NRP-4 family.

**Figure 3:**
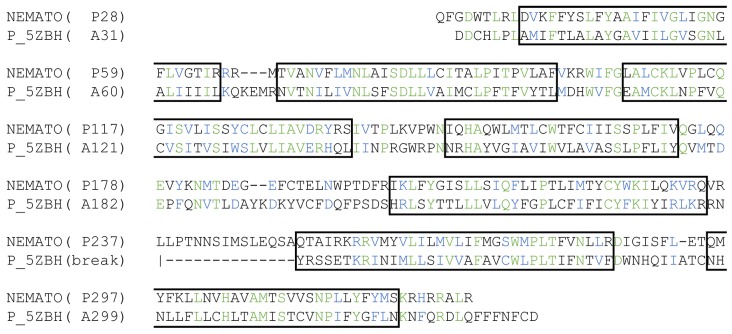
Sequence alignment used for the homology modelling: alignment between Minc3s00007g 00462 (NEMATO) and the human neuropeptide Y Y1 receptor (PDB structure 5ZBH [33]). Identical residues are in green, similar residues are in blue and the helices sequences in black rectangles.

Consequently, we selected the PDB template 5ZBH to build the 3D model of Minc3s00007g00462 and the two proteins sequences were aligned according to their similarities and the position of their transmembrane helices (Figure 3).

### 2.3. Molecular Dynamics Simulations

**Figure 4:**
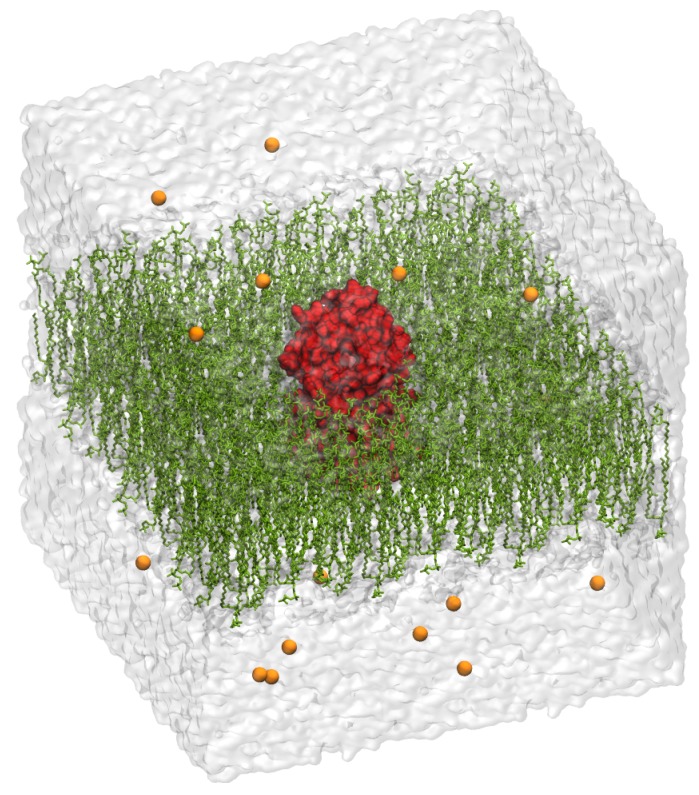
Appearance of the initial Model 3. The red colour indicates the GPCR, the olive green colour indicates the membrane lipids, chloride counterions are represented in orange and the grey colour indicates the water box.

**Figure 5:**
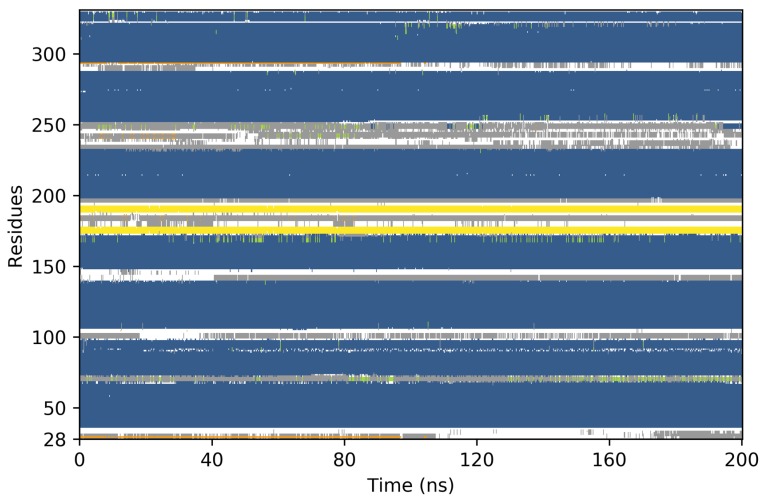
Evolution of the secondary structure during the 100 ns Molecular Dynamics (MD) (blue: α-helices, yellow: β-sheets, green: 3${}_{10}$ helices, gray: turns, white: coils).

The 3D model of Minc3s00007g00462 is presented in Figure 4. Concerning the 3D model conformational behaviour, the main focus was firstly the seven transmembrane helices: if one of them broke permanently, the model was not considered as stable and a new model had to be built from a new alignment. During the first 10 ns, all helices were stables. The MD simulations were prolonged to 100 ns and all helices remained stable except for the second helix which broke and reformed itself frequently (Figure 5). This deformation can be explained by the presence of two prolyl residues at position 91 and 94 that destabilized the helix. As the helix frequently reformed itself during the simulation, we considered the model as stable during the MD simulations.

When observing the variation of the RMSD of the GPCR backbone during the MD (taking the first frame as the reference), the largest values (up to 8 Å) came from the loops connecting the TM helices (especially the ones which should be intracytoplasmic). In contrast, the TM7 helix bundle and the small β-sheet found in the large loop connecting TM4 and TM5 remained stable (maximum RMSD of 2.5 Å and 0.8 Å respectively). The behaviour of the receptor indicated that the model was robust enough to be used later for our virtual screening campaign.

### 2.4. Selection of the Conformational Ensemble for the VS Campaign

**Figure 6:**
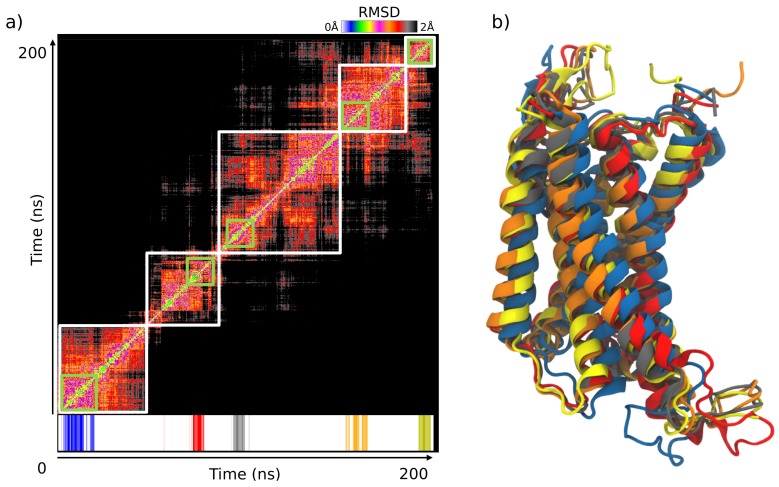
((**a**), top) RMSD map. The five retained conformation families are represented in white; ((**a**), down) The top five Self Organizing Maps (SOM) clusters containing the largest number of frames (represented in green) are superimposed on the RMSD map. (**b**) Structural alignment with a 3D cartoon representation of the five selected protein conformations retained to perform the docking campaign (blue 24 ns, red 68 ns, grey 130 ns, orange 170 ns, yellow 190 ns).

From the 200 ns MD simulations, according to the RMSD analysis, it appeared that the molecular system adopted five families of stable conformations (Figure 6a), c1 to c5, respectively around 24 ns, 68 ns, 130 ns, 170 ns and 190 ns. This analysis was supported by the SOM clustering as shown on Figure 6. By considering only the 5 most dense clusters obtained after SOM, these SOM clusters present similar sizes and fit well in the regions delimitated by the RMSD map. The alignment of the 3D structures representing each of the five families are presented in Figure 6b. The RMSD between the five groups were always between 1.8–4.8 Å.

**Figure 7:**
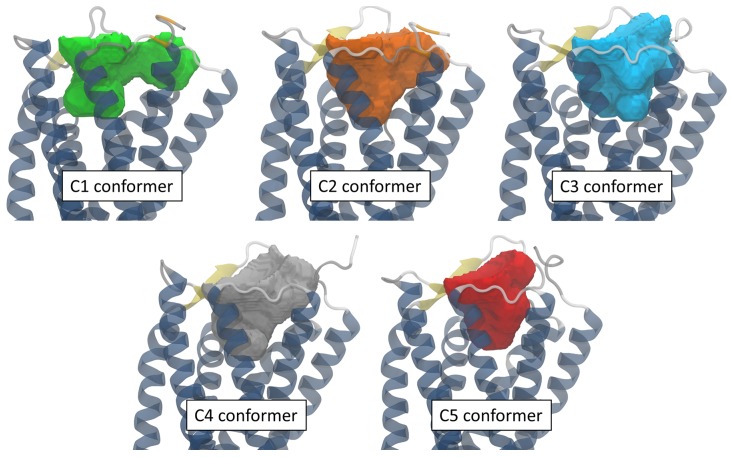
Shape and position of the binding pockets obtained for the 5 MD conformers retained for the ensemble docking with the 3V web server [34].

The observation of the binding pockets for each of these conformers revealed that all the pocket shapes differ due to different side chains orientation of the residues lining the binding site despite roughly similar values obtained for their volumes (around 2000–2500 Å${}^{3}$) and surface areas (around 1200–1300 Å${}^{2}$) (Figure 7). This would have consequences on the docking results and such a situation highlight the interest of using an ensemble docking program such as GOLD.

### 2.5. Virtual Screening Campaign and Compounds Selection

From the docking campaign using these five main protein conformers, we retained the top 100 molecules from the complete PLP score list for further analysis. From this list, it appeared that 85 compounds came from the ChemDiv provider’s library. Hence, for consistency, we kept only these 85 compounds in the rest of our analysis.

After inspection of molecular weights, solubility (we discarded all compounds with a logP > 5.00), compound similarities and toxicity, 13 molecules were finally selected (Table 1). For example, compounds with docking scores ranked 1 to 9 and 11 to 14 had bad solubility scores >5.00 and were removed. Consequently, the first high-scoring molecule retained was ranked only 10 in the top 100 list.

**Figure 8:**
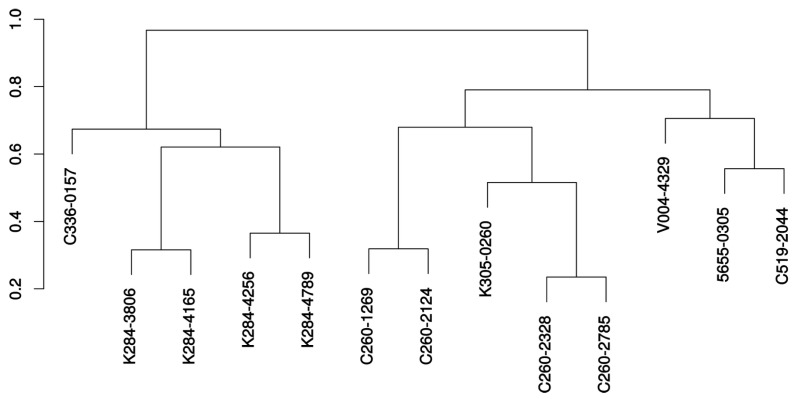
Dendrogram showing the clustering of the 13 retained molecules. The dendrogram was built with Ward’s method using Tanimoto scores.

**Figure 9:**
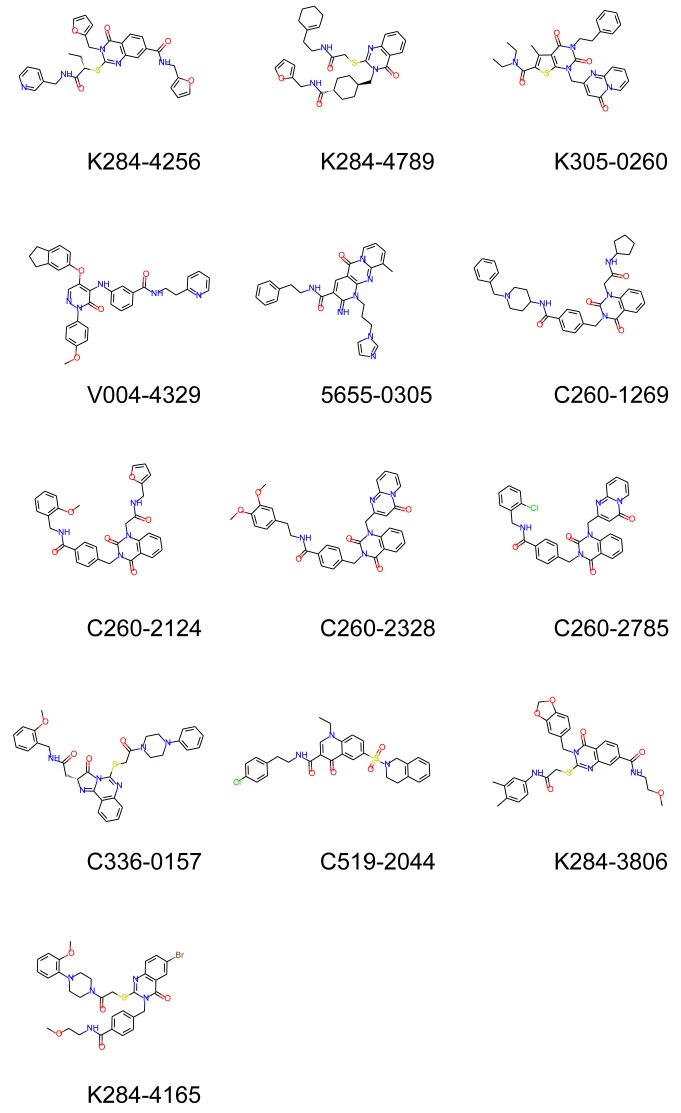
Molecular formula of the 13 selected molecules.

A dendrogram representing the chemical relationship between the 13 retained compounds is presented in Figure 8 and the molecular formula of these molecules are shown in Figure 9. It is seen from this dendrogram that the retained compounds could be roughly classified into two groups, each presenting two subgroups.

**Figure 10:**
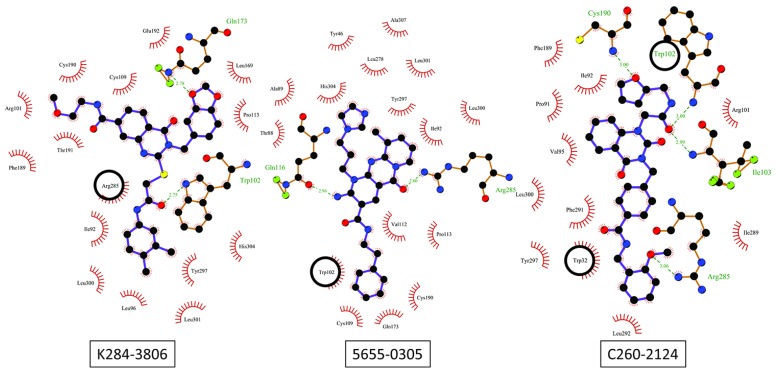
LigPlot showing the diversity of protein/ligand interactions between 3 retained compounds, respectively bound to c2, c3 and c5 protein conformations. The black circle corresponds to π-π interactions.

Looking at the interactions of these 13 compounds within the protein binding pocket, 12 retained ligands are found mostly bound to the c3 or the c2 conformations (9/13 and 3/13 respectively). The only exception was the C260-2124 compound that gives its highest score when bound to the c5 protein conformation. Considering hydrogen binding interactions, a large variety of binding possibilities were found, but the only residues that bind the majority of retained compound through H-bonds (or -stacking) are Trp102 and Arg285. All other interactions were specific to one or two ligands. We illustrate in Figure 10 the ligand-receptor interaction differences between the 5655-0305 compound as bound to the c3 conformation, the K284-3806 compound bound to the c2 conformation and the C260-2124 compound bound to the c5 conformation.

### 2.6. Bioassay

**Figure 11:**
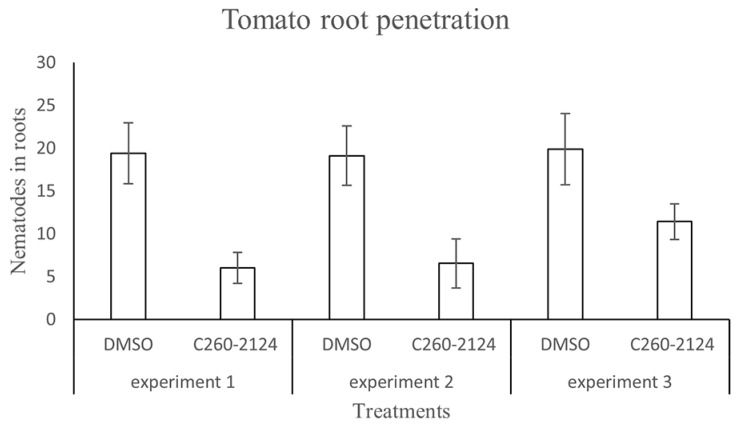
Number of *M. incognita* larvae in tomato roots inoculated with DMSO- treated J2s (control) or C260-2124-treated J2s in three independent experiments. Bars indicate mean number of nematodes per plant (n = 30) and error bars indicate the standard error (SE) of the mean. Penetration was recorded after 3 days.

The 13 candidate molecules, retained from the virtual screening list above, were tested in nematode assays. Direct exposure of nematode infective juveniles (J2s) to the selected compounds at 1% did not affect their viability. Nevertheless, in preliminary qualitative tests in pluronic medium (supp figs), the observed RKNs movements seem to be altered, probably because of the drug interaction with the predicted nematode chemosensor receptor target. Even if reduction effects on the penetration of J2s in plant roots were observed with several compounds, reliable and consistent results of treated nematodes were only obtained with the C260-2124 compound in three independent experiments (Figure 11). We observed a reduction of 40 to 60% in nematode penetration in tomato roots when nematodes were treated with the C260-2124 compound as compared to DMSO controls. This result indicates no interference of the drugs’ solvent DMSO in the control assay.

## 3. Discussion

**Figure 12:**
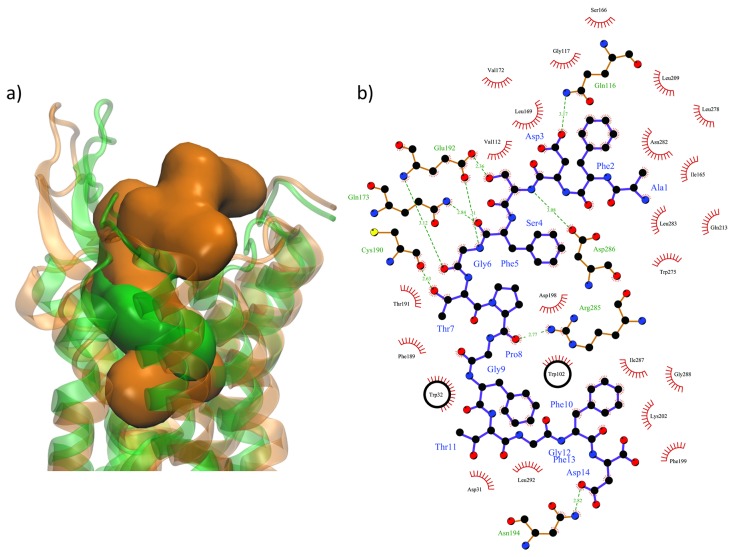
(**a**) Respective molecular surfaces of the NLP15 peptide (in orange) and our active C260-2124 compound (in green) inside the binding pockets lining the TM7 bundles of the nematode GPCR in the c3 and c5 conformations respectively (in transparencies with corresponding colours with the ligands); (**b**) Ligplot diagrams showing the interactions between the peptide compound and the nematode GPCR. TRp102 is implied in π stacking interactions, and Arg285 is engaged in H-bonds. Black circles represent π stacking interactions.

Searches for novel nematicides have been tackled starting mostly from profiled peptides belonging to the FMRF-amide or to the neuropeptide families. FMRF-amide like peptides represent the most widely investigated family. They are characterized by -RFamide motif and are known to be involved in motor and sensory function coordination with effects on nematodes infectivity [16]. The neuropeptide related compounds are known to dysregulate the normal behaviour of penetration while little is known about their function in nematodes. Nevertheless, recent studies concerning such neuropeptides-like proved to dysregulate key behaviours [24], especially in *M. incognita* [35]. The most active of such peptides has the AFDSFGTPGFTGFD sequence therefore presenting four aromatic side chains so that it is interesting to compare the docking result of this peptide in our *M. incognita* GPCR models with the one obtained for our C260-2124 compound. Such comparison is shown in Figure 12a where the two compounds share the same part of space inside the 7TM helices binding pocket and with similar interactions, especially H-bonds and stacking (Figure 10 and Figure 12b). Besides the two Arg285 and Trp102 residues already implied in the interactions with our 13 selected compounds, several other residues where found interacting with both the AFDSFGTPGFTGFD peptide and the C260-2124 compound, such as Cys190 from its backbone carbonyl and Trp32 through π stacking of its indole ring with aromatic moieties of the ligands. This result reinforces the idea that C260-2124 can be considered as a valuable hit for further investigation to lead drug design studies.

**Figure 13:**
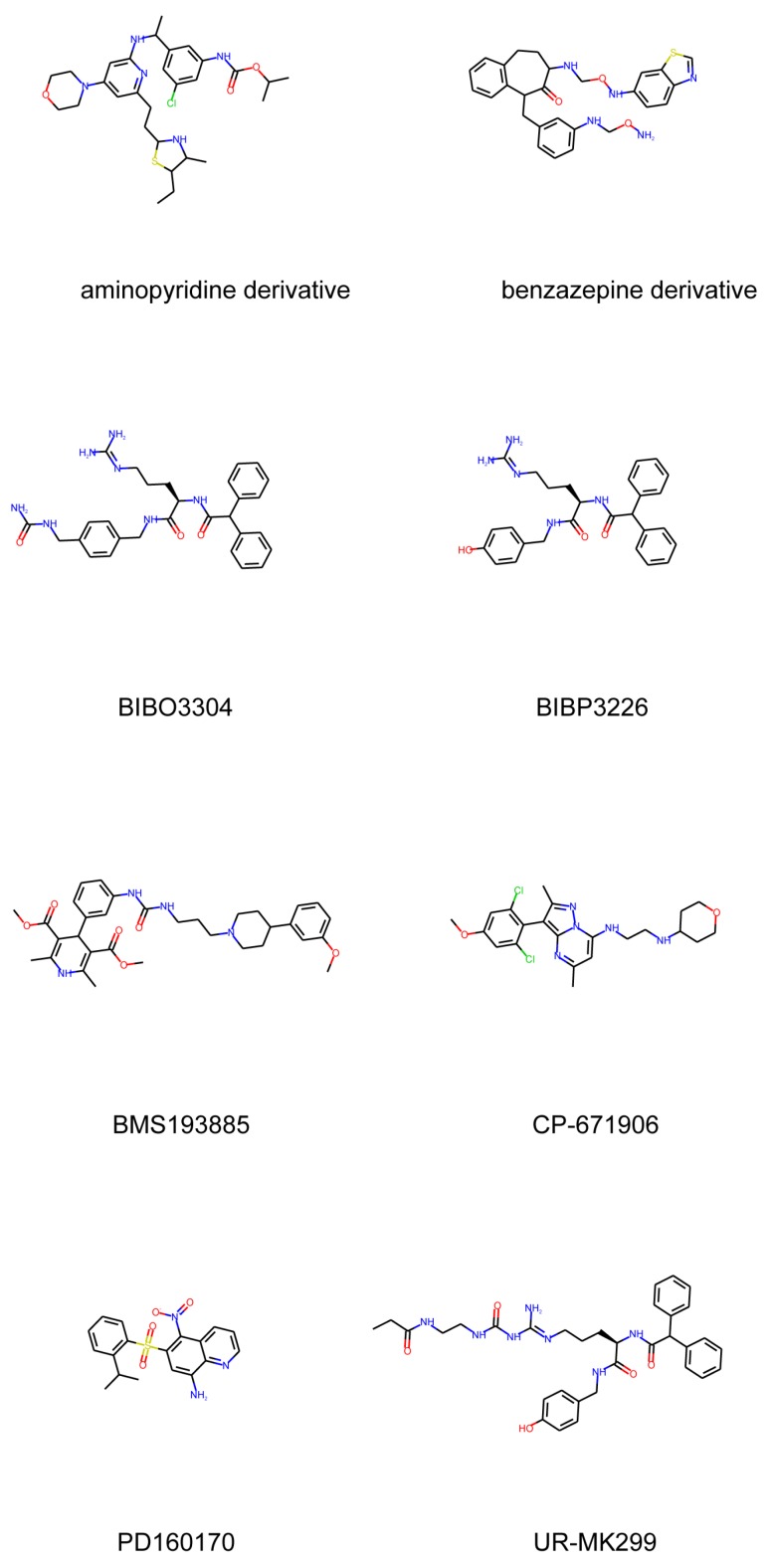
Low molecular weight organic compounds antagonists to NPY Y1R that were evaluated with the GOLD docking procedure hereby described.

Apart from peptide-related compounds, few small molecular weight organic compounds were also reported in the literature. Besides BIBP-3226, which was the first selective neuropeptide Y1 receptor (NPY Y1R) antagonist found in 1996 [36]. Some of the proposed compounds were based on: benzazapine nuclei [37], phenoxyquinlines [38], substituted alkoxy-aminopyridine groups [39], pyrazolo-pyrimidines fragments [40] and pyridinedicarboxylic acid derivatives such as BMS-193885 [41], on carbamoylation of argininamide moities such as UR-MK299 [42]. Recently, the two last antagonists, namely UR-MK299 and BMS-193885 were used in the X-ray study presenting the 3D structure of the NPY Y1R in complex with these molecules. Figure 13 lists and presents the chemical formulas of these compounds.

Docking of all these compounds according to a process similar to the one followed in our virtual screening shows that only the UR-MK299 compound gave a score in the range of the ones obtained with our 13 selected molecules. When looking at the interactions stabilizing this ligand, it appears that, besides several H-bonds implying the backbone of Glu192 and Cys109, as well as the sidechain of Gln116, the two residues Arg285 and Trp102 found as stabilizing all our 13 compounds through H-bonds or π-stacking, are also engaged in similar interactions. Such result highlights the role of these two last residues in the recognition of antagonist compounds.

**Figure 14:**
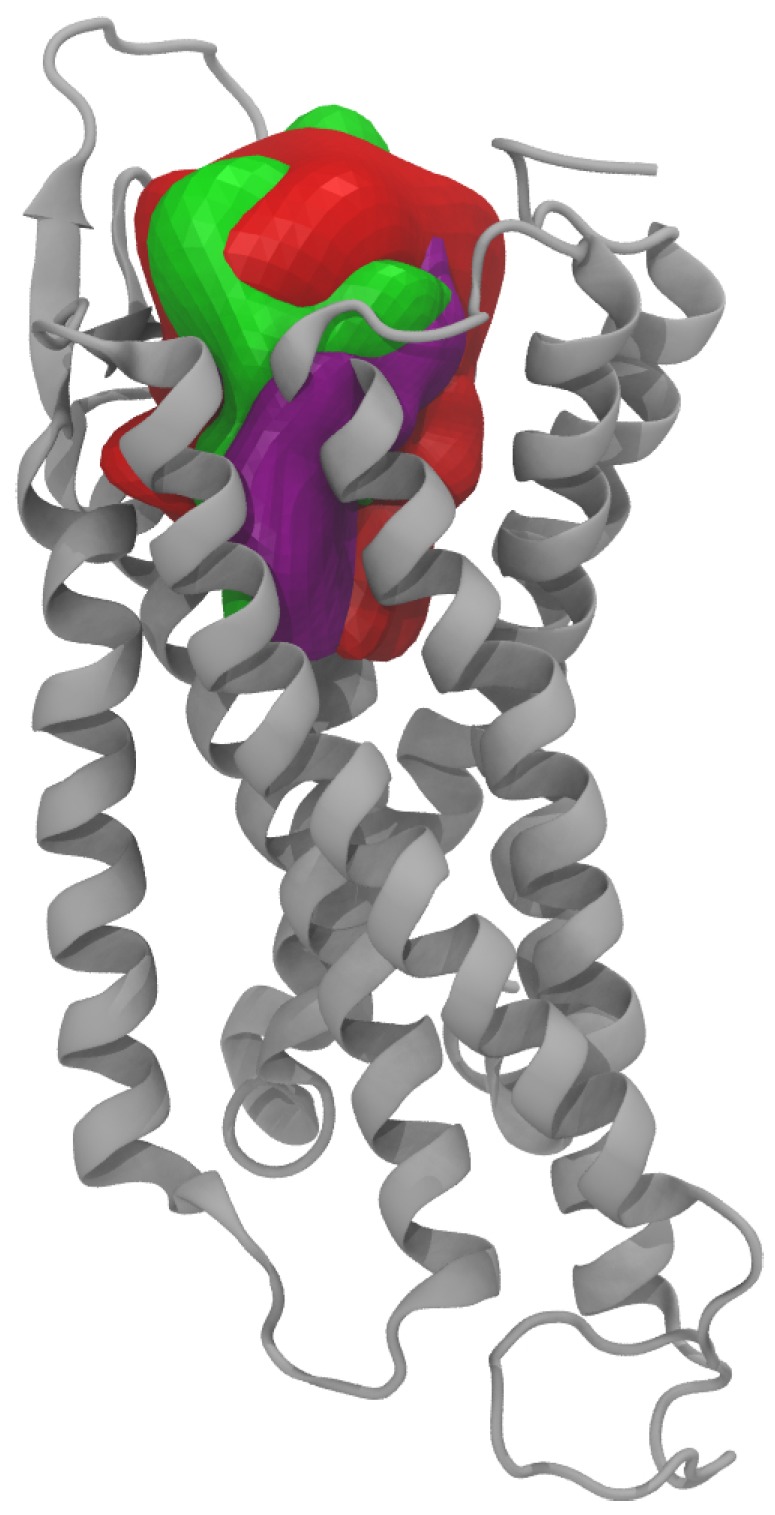
Binding cavity surfaces (in red) compared to the ones obtained in the PDB 5ZBH (in green) and 5ZBQ (in purple) within the GPCR 7-helices bundle (in gray).

Because we used the human neuropeptide Y Y1 receptor (NPY Y1R) as a template, the question of the specificity of the compounds identified in our study to the nematode and their possible side effects must be considered. To check that, it is interesting to compare the protein/ligand binding characteristics found in the Y Y1 neuropeptide receptor template versus our model. When looking at the ligands chemical structures and binding properties found in the 5ZBH and 5ZBQ PDB structures and comparing them to the 13 proposed compounds from our virtual screening, it appeared that:

- The binding cavity obtained for our nematode GPRC receptor presented a quite similar 3D shape when compared to the ones obtained for the two 5ZBH and 5ZBQ X-ray structures (Figure 14): calculated cavity surface area and cavity volume of 913 Å${}^{2}$ and 1572 Å${}^{3}$, in the range of the ones obtained for the two X-rays: 1067 and 870 Å${}^{2}$ respectively and 1806 and 1352 Å${}^{3}$ respectively;
- The two inhibitors (UR-MK299 and BMS-193885 respectively) complexed to the neuropeptide Y Y1 receptor have very different chemical structures when compared to our selected compounds with Tanimoto coefficients less than 0.3;
- In our model most of the 13 compounds interact through H-bonds with Arg285 (which is not conserved in the Y Y1 receptor) and with Trp102, Gln116 and Gln173 (which are conserved residues between the two species, corresponding respectively to Trp106, Gln120 and Gln177 in YY1). In the Y Y1 3D structures, only interactions with Gln120 are similar to the ones found with our compounds. Gln120 is proposed to be crucial for receptor activation as being the interaction partner for the NPY peptide C-terminus.
- Additionally, interactions are also found in our model with residues Tyr46 and Trp102 (both conserved in Y Y1 as Tyr47 and Trp106 respectively, but none of these two residues were found to interact with the inhibitors in the X-ray structure of YY1. Supplementary cation interactions implying Lys100 and Arg285 (both not conserved) are also stabilizing the interactions with some of our 13 molecules. It could be concluded from this analysis that, despite a quite similar binding site compared to the ones in human YY1 receptor, our compounds should be very specific to neuropeptide nematode GPCRs with few or no interactions with human neuropeptide receptors, so that no unwanted effects can be expected.

## 4. Materials and Methods

**Figure 15:**
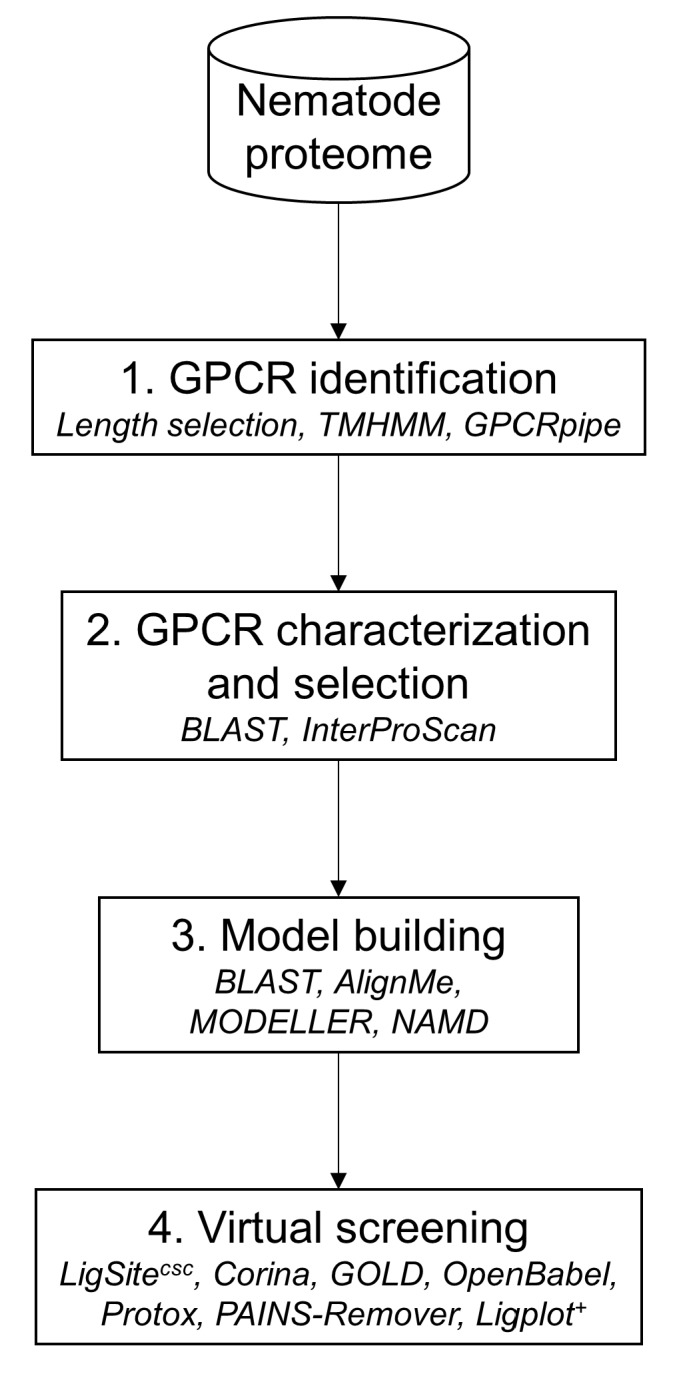
Data analysis framework for prediction of GPCRs. Used programs are in italics.

The in silico approach was composed of four main steps as illustrated in Figure 15 (more details regarding the methods please see Supplementary Materials). The first step was to filter the proteomic dataset from the nematode to identify GPCRs using several filters (sequence length selection, transmembrane helices prediction and GPCR prediction tool. Next, the GPCRs candidates were characterized in order to select a limited number of possible targets using sequence similarity. The third step consisted to build a 3D model of the selected GPCR candidate and to validate it by a 100 ns molecular dynamics simulation. The last step was to submit the GPCR candidate stable model to the virtual screening in order to identify probable molecules to inhibit the target.

### 4.1. GPCR Identification in Nematode Genome

The initial dataset used for GPCRs prediction comprises *M. incognita* recent genome annotation available V2 [43]. As the majority of GPCRs have a length between 250 and 1000 residues, the first step of our pipeline consisted in filtering proteins within the predicted proteome according to this range. The main GPCRs signature is their seven transmembrane helices so the retained proteins were submitted to TMHMM [44] in order to determine their number of transmembrane helices. Finally, GPCRpipe [45] was used on proteins with seven transmembrane helices to predict GPCRs. GPCRpipe was configured to “and” method, meaning that a protein was predicted as a candidate if GPCRpipe HMM and Pfam 3915 profile HMM predicted it as a GPCR. GPCR class was predicted with InterProScan [46].

### 4.2. Homology 3D Model Building

Although automatic methods for GPCR homology modelling were recently developed [47,48], the construction and validation of the various homology models of GPCRs is still a challenge [49] and requires not only the classical multiple sequence alignments but would also include structure-based alignments. This approach has been proved successful in many studies [50,51]. Nowadays, the structure of 20 different Class A, two Class B, two Class C, and one frizzled GPCRs are available in the PDB [52], providing a reasonable set of possible templates to be used.

For the template choice, selected GPCRs were aligned by PSI/TM-Coffee [53] with PDB GPCRs. The phylogenetic analysis was done with phylogeny.fr web service [54]. After alignment, ambiguous regions (i.e., containing gaps and/or poorly aligned) were removed with Gblocks [55]. The phylogenetic tree was built using the maximum likelihood method implemented in the PhyML program [56,57]. Reliability for internal branch was assessed using the aLRT test (SH-Like). Graphical representation and edition of the phylogenetic tree were performed with iTOL webserver [58].

To build the model, the selected protein and the template were aligned with AlignMe [59], a dedicated program for membrane protein alignment, taking into account sequence properties and secondary structure predictions. The model was built with MODELLER with the automatic loop refinement method [60].

### 4.3. Molecular Dynamics

The next steps using Molecular Dynamics (MD) simulations were required to refine the preliminary crude model and then analyse the stability of the GPCR within the membrane. For that, we used the NAMD molecular dynamics software [61]. MD is now commonly used to validate homology models, especially in the GPCRs field [62,63,64,65].

For this purpose, we used molecular dynamics simulation on the receptor homology models that were embedded in a fully hydrated POPC bilayer [66]. No ligand was positioned within the receptor at this level as it has been shown [67] that the presence of a ligand does not change the accuracy of the structure produced. Initially, the receptors models were positioned across the equilibrated bilayer while seeking to match the hydrophobic protein segments with the layer formed by the lipid hydrocarbon tails. Lipids overlapping with the protein complex were deleted, leaving a bilayer consisting of 584 POPC molecules. To ascertain that the cytoplasmic and extracellular loops did not interact, an amount of 79,089 water molecules was added, as well as 16 chloride counter ions to make the whole system-neutral, thus making a total number of atoms equal to 328,547. The complete system, represented in Figure 4 was replicated periodically in the three directions of space, with a repeat distance of ≈130 Å.

The MD simulations were carried out in the isobaric-isothermal ensemble, maintaining the pressure and the temperature of 1.0 atm and 300.0 K, respectively, using Langevin dynamics and the Langevin piston approach. The MD program NAMD was employed in conjunction with the CHARMM27 force field [68] to describe the receptor, the lipid bilayer, and the water molecules. Coulomb forces were evaluated with the particle-mesh Ewald method. The equations of motion were integrated with a 1 fs time step, using the r-RESPA algorithm to update short- and long-range contributions at different frequencies.

Each system was energy minimized and then equilibrated before recording trajectories. All MD trajectory frames were recorded at 1 ps intervals, for a total of 10 ns simulation. Model stabilities were then checked by analysing secondary structure evolution during the MD simulation. If at least one transmembrane helix broke, then the model was not considered stable. For the stable models obtained, the simulations were extended to reach a 100 ns simulation time.

### 4.4. MD Simulation Analysis

Once the MD simulations finished, all the frames were aligned on the first frame to calculate RMSDs with VMD (“RMSD trajectory tool” plugin) [69]. An RMSD map was built by a previously developed in-house TCL script.

In addition to the RMSD map, the trajectory obtained after molecular dynamics was analyzed by the Self Organizing Maps (SOM) method [70]. The method consisted in transforming the coordinates of each frame using a Principal Component Analysis (PCA) and superimposing them on a neural network used to separate the frames [71]. Similar frames were found in the same cluster. To validated this classification, the SOM method was repeated 30 times and Rand index was calculated for each run. The HGPA (Hyper-graph partitioning algorithm) cluster aggregation method is used to converge to a consensus [72] between these clustering steps.

### 4.5. Virtual Screening

The initial dataset of chemical libraries were the GPCR-centred libraries encompassing six compounds providers: Asinex [73], Chembridge [74], ChemDiv [75], LifeChemicals [76], Otava [77] and Selleckchem [78], representing 112,951 molecules. The 3D structures of all the molecules were obtained from the Corina software [79]. The compound protonation states and atom names were corrected if needed, respectively, for pH 7 and compatibility with the GOLD program.

The docking was performed by GOLD which has been recognised as one of the best docking softwares [80,81]. As several stable conformational families were identified, the ensemble of docking possibilities was used. The use of such conformational ensembles was considered as an improved strategy in structure-based docking calculation [82]. For each docking, 50 starting ligand conformers were used in GOLD. All target conformations were aligned on a common reference system and the centre of the binding pocket as an average of the individual centres determined by LigSite${}^{csc}$ in each conformation [83]. The binding region was defined as a 15Å radius sphere around this centre. Each docking was evaluated by the piecewise linear potential (PLP) scoring function for protein-ligand docking [84,85].

In order to avoid possible toxicity, the candidate compounds were surveyed using predictors such as PAINS-remover [86] and ProTox web service [87]. The structural similarity of the selected compounds was evaluated by the Tanimoto index [88,89]. The score was calculated by Open Babel using FP2 fingerprint [90]. These similarities were represented as a dendrogramm created by hierarchical clustering with Ward’s aggregation method. For the retained compounds, protein-ligand interactions were detected by PLIP [91].

### 4.6. *In Vivo* Experiments

As it was not presently possible to perform in vitro tests to directly measure the binding of our selected compounds to the GPCR receptor, we will present here only very preliminary in vivo experiments. Robust protocols for in vivo testing of products acting on root-knot nematodes are difficult to handle and are time consuming. Valuable experiments are not as easy as for other systems because of the particular surrounding conditions necessary for establishing nematode growth. Furthermore, as an obligatory endo-parasite of plant roots, the only life stage that can be used for testing attack inhibition is the infective juvenile stage [92].

#### 4.6.1. Nematode Culture Preparation

*Meloidogyne incognita* was obtained from pure cultures maintained in roots of tomato plants (*Solanum lycopersicom*, var. Santa Cruz Kada)). Tomato seeds were surface sterilized with 1.4% NaOCl for 5 min followed by 2 × 15 min rinse in sterile water. After 7-days germination in Germitest paper, tomato seedlings were transferred to a plastic pot filled with a sand/hydrogel mixture (3 seedlings in 5-mL substrate per pot). To perform the mobility test nematode second-stage juveniles (J2) were obtained from *M. incognita* egg masses extracted from previously infected plants. After approximately two reproductive cycles, the nematode eggs were bulk extracted from severely infected tomato roots with 0.05% (v/v) sodium hypochlorite (NaOCl) as previously described [93]. The J2 were subsequently washed with distilled water and left for hatching in sterile water for 3–5 days at 25 ${}^{\circ }C$ using the Baermann funnel method [94]. Freshly hatched J2s were counted and used for all bioassays.

#### 4.6.2. Compounds Preparation

Compounds (2–5 mg each) were suspended in DMSO solution to reach a 10 mg mL${}^{-1}$ concentration.

#### 4.6.3. Bioassays

Tomato roots were inoculated with *M. incognita* J2s previously treated in vitro (eppendorf) with each selected compound diluted to 1% with distilled water (v/v stock compound). Worms treated with DMSO 1% were used as control. To verify the inoculum viability after 24 and 48 h of exposure direct exposure to the diluted compounds, the J2’s motility was observed over a microscope slide under visible light (Leica). The in vivo validation of the selected compounds was performed by measuring the nematode ability to penetrate tomato roots when treated with the compounds. This ability was tested with exposure to compounds at 1% concentration by counting the number of J2s in roots at 3 days after inoculation (dai). A total of at least 300 freshly hatched J2 were added to each pot. The whole experiments contained 30 replicates per treatment. The inoculated plants were cultured at room temperature with 16h/day natural light. The *M. incognita* J2 penetration rate was recorded after 3 dai by observation under a stereo-microscope (Leica) of fuchsin-stained roots [95].

## 5. Conclusions

The root-knot nematode *M. incognita* is one of the most aggressive and damaging plant-parasitic nematodes as it uses a sophisticated chemosensory apparatus to detect potential hosts and to infect them. Chemical nematicides have so far constituted the most efficient tools against these agricultural pests. Because of their toxicity for the environment and danger to human health, most nematicides have now been banned from use. Consequently, new and more specific means of control, which are safe for the environment and human health, are urgently needed to avoid worldwide proliferation of these devastating plant-parasites. Understanding the parasite machinery, aiming at identifying novel targets for the development of future control methods is an important issue that concentrate the efforts of many research groups. For that purpose, three main lines of action can be followed dedicated respectively to the detection of the hosts by the parasite, its penetration within the host roots, and the development of the giant cells within the roots. We focused our study on the role of GPCRs possibly involved in the mechanism of parasite detection and locomotion towards its host’s roots. Among all the putative GPCRs genes in the *M. incognita* genome, we found one GPCR belonging to the neuropeptide receptors family that appeared as an interesting target against *M. incognita*. A drug design strategy merging *in silico* and experimental bioassays was successfully undertaken in order to find new low molecular weight chemical compounds able to interfere with this GPCR.

**Figure 16:**
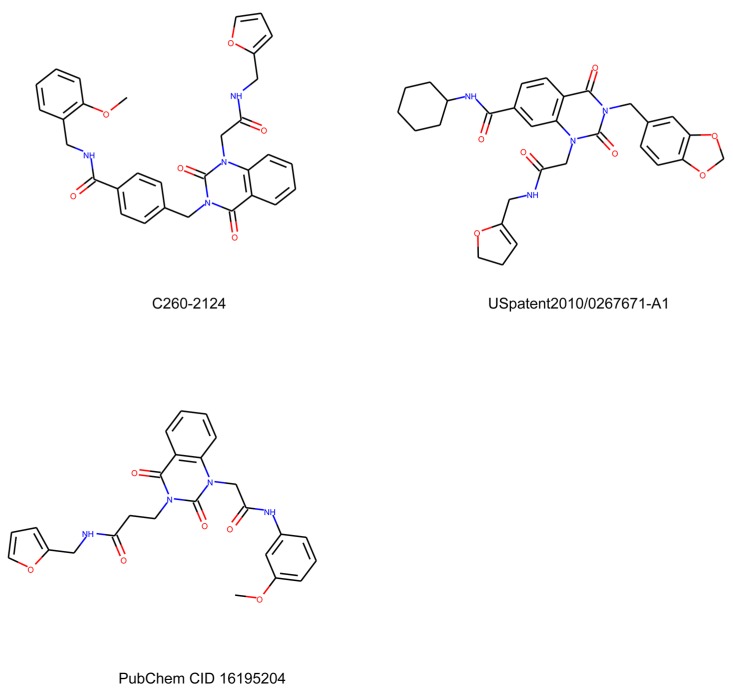
Chemical structure of our compound C260-2124 versus the two closest molecules found and already used in bioassays.

The data obtained in the present study show that some of the tested compounds affect mostly or only the penetration rates most probably due to muscle contraction/behavioral disruption [96,97]. These results suggest that some of these substances, although not affecting mortality, may change the nematode behavior and its sigmosoidal crawling [96]. Among these tested compounds, C260-2124, was found as presenting the required anti-nematode characteristics. After examination of chemical literature and patents data, this compound is not referenced for any biological assays. Only few similar compounds were found presenting some biological action. The two closest ones (Tanimoto index around 0.7) are presented in Figure 16: the first one is described in PubChem (MLS00073044 and CID 16195204) and was tested in bioassays against the protein target huntingtin (Huntington disease) and the second compound was described in US patent 2010/0267671A1 to modulate apoptosis in cells.

Our biological assays were preliminary and further investigations now have to be performed to definitively validate our results and investigate the specificity and safety of this compound for non-target organisms. These preliminary studies indicates the targeting of some of the selected compounds against the nematode neuromuscular system as previously pointed by Kumari et al. [16]. However, due to the complexity of experimental conditions, a complete molecular characterization was not performed in this study. We report here only potential compounds that affect important roles in the *M. incognita* chemoreception of environmental stimuli as one of the major sensory system. These findings may allow further research, innovation and development. Nevertheless this work is the first one proposing a non-peptide compound acting as an antagonist of *M. incognita* neuropeptide receptor, therefore validating our design strategy. C260-2124 can be considered as a good seed to further develop new leads and this work demonstrated the strength of combining in silico and experimental approaches.

Moreover, all the putatively C260-2124-binding amino acid residues of Minc3s00007g00462 are conserved in the homologous sequences of the other RKN of major concern *Meloidogyne hapla*, *M. javanica* and *M. arenaria*. This data suggest that C260-2124 could also be active on these others nematode species of high economic interest.

This work participates in the efforts of the research community to identify new classes of nematicides and we believe this approach will help to strengthen the present arsenal. Besides recent screening approaches mostly conducted on the *C. elegans* nematode [98,99], our approach demonstrates that a GPCR involved in the parasite “GPS”, guiding it towards plants roots, can also be a valuable target for developing innovative compounds.

## Figures and Tables

**Table 1:** Molecules tested in bioassays with nematodes.

Compound ID	MolWeight	logP	Rank
5655-0305	481.56	1.38	18
K284-4256	557.63	2.37	28
C260-2124	552.59	3.13	69
V004-4329	573.66	3.43	23
C260-2328	617.67	3.7	97
K305-0260	543.65	3.8	42
C336-0157	596.71	3.87	20
C260-2785	578.03	4.07	83
K284-3806	574.66	4.27	15
C519-2044	550.08	4.43	27
C260-1269	593.73	4.45	10
K284-4165	680.63	4.52	17
K284-4789	562.74	4.79	12

## Supplementary Materials

The following are available online, Supplementary methods, Supplementary results, Figure S1: *Meloidogyne incognita* J2 at the apical zone of the tomato root meristem, Figure S2: *Meloidogyne incognita* J2 in fuchsin staining nematode in root tissue.
