# Supplementary material for: A Chemosensory GPCR as a Potential Target to Control the Root-Knot Nematode Meloidogyne incognita Parasitism in Plants
Source: Molecules. 2019 Oct 22;24(20):3798. doi: 10.3390/molecules24203798 (PMC6832152; doi:10.3390/molecules24203798)
Supplement: Supplementary file 1 [file molecules-24-03798-s001.pdf]

# Supplementary Materials: A Chemosensory GPCR as a Potential Target to Control the Root-Knot Nematode *Meloidogyne incognita* Parasitism in Plants

**Emmanuel Bresso, Diana Fernandez, Deisy X. Amora, Philippe Noel, Anne-Sophie Petitot, Maria-Eugênia Lisei de Sa, Erika V. S. Albuquerque, Etienne GJ. Danchin, Bernard Maigret and Natália F. Martins**

## 1. Supplementary methods

### *1.1. Pluronic acid gel preliminary studies*

The behavior of J2 nematodes was observed under acid pluronic F-127 media in the presence of selected chemical compounds. Under experimental conditions the J2 would freely move towards the tomato roots. The transparency allows the observation and the performance of behavioral assays [97].

### *1.2. Plant preparation*

Seeds of the tomato cultivar 'Santa Cruz Kada Gigante' were sterilized and germinated over a sterile filter, humidified in Petri dish, in the dark, under environmental temperature.

### *1.3. Nematodes*

The inoculum of *M. incognita* was obtained from a nematode population culture maintained on tomato 'Santa Cruz Kada Gigante', cultivar grown in greenhouse. The freshly emerged J2s were counted under the stereomicroscope and used in the behavioral assays.

### *1.4. Pluronic gel preparation*

23 g Pluronic F-127 powder (NF Prill Poloxamer 407, BASF, Mt Olive, NJ, USA) was added to 80 mL cold, sterile water at 4 °C and allowed to dissolve with stirring for 24 h. The dissolved gel was stored at 15 °C and aliquots were dispensed for behavioral assays.

### *1.5. Behavioral assay*

Each candidate compound was diluted in DMSO at 1% (1µg/100µL). 20 mL of 23% Pluronic gel containing poured into each Petri dish at 15 °C. A suspension containing 800 J2/2mL was incubated with 80 microliters of the candidate compound for 10 minutes at room temperature. The mixture was added to the pluronic gel in a petri dish. After homogenization, 2.0 mL of the J2 suspension was poured in triplicate in 3.5 cm diameter petri dish. Tomato sterile seedlings were added to the dish and placed at the gel at room temperature to solidify. The behavior of J2 was recorded in 12 hs and 24 hs after incubation in a Leica M205 FA.

The ability of nematodes to infect tomato roots was evaluated after 48h with fuchsin staining method according to Bydb *et al.* (1983) [92].

## 2. Supplementary Results

Nine compounds were tested in comparison to 1% DMSO used as control and in triplicates. In water and DMSO, J2 showed the typical sinusoidal movement towards the apical meristem of tomato roots. After six and 12 hours we observed the accumulation of J2 in the root apical meristem. In the control, the majority of J2 had penetrated after 24 h of incubation.

In the presence of compounds K284-3806 and V004-4329 J2 movements were observed to be atypical, presenting both strengthen movements, slowly for those alive; additionally the majority of J2 were not moving in the gel (Figure S1). Very few juveniles were observed in the root apical meristem and at the fuchsin staining method (Figure S1)

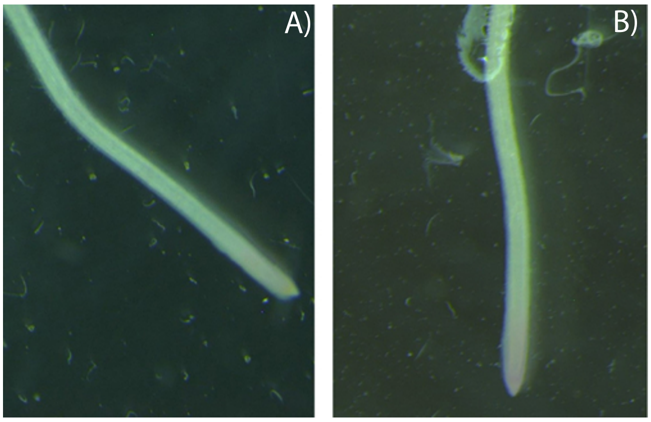


**Figure S1.** *Meloidogyne incognita* J2 at the apical zone of the tomato root meristem. A) Incubation in water. B) incubation with compound K284-3806 after 24 hours showing that the number of nematode is lower in B than in A.

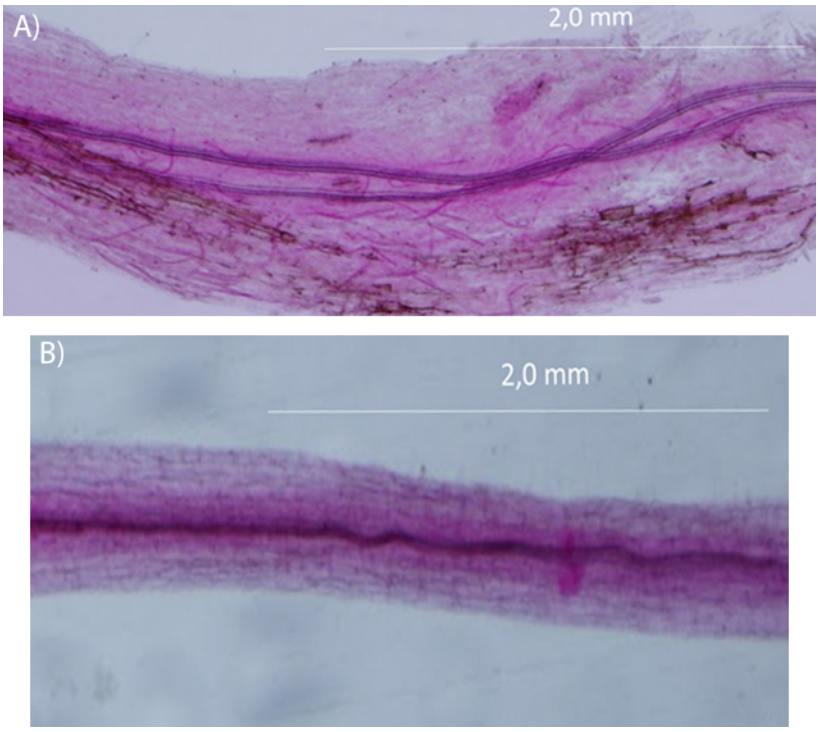


**Figure S2.** *Meloidogyne incognita* J2 in fuchsin staining nematode in root tissue. A) Incubation in water. B) Incubation with K284-3806 showing that the number of nematode is lower in B than in A.
